# Starting fresh: a mixed method study of follower job satisfaction, trust, and views of their leader’s behavior

**DOI:** 10.3389/fpsyg.2024.1349353

**Published:** 2024-02-16

**Authors:** Paul E. Spector, David J. Howard, Eric M. Eisenberg, John D. Couris, Joann F. Quinn

**Affiliations:** ^1^People Development Institute, Tampa General Hospital, Tampa, FL, United States; ^2^Muma College of Business, University of South Florida, Tampa, FL, United States; ^3^Executive Offices, University of South Florida, Tampa, FL, United States; ^4^Hospital Administration, Florida Health Sciences Center, Tampa General Hospital, Tampa, FL, United States; ^5^College of Medicine, University of South Florida, Tampa, FL, United States

**Keywords:** follower experience, job satisfaction, leader behavior, leadership, leader trust, mixed method

## Abstract

**Introduction:**

The leadership literature has been dominated by the study of broad styles rather than the identification of specific key behaviors. To address this deficiency, a mixed method approach was utilized to explore how follower behavioral descriptions of their leaders would relate to potential outcomes of trust in that leader and job satisfaction.

**Methods:**

Data were collected from 273 hospital direct reports of 44 managers. They were asked to first describe the leadership approach of their managers in their own words, and then complete quantitative measures of the two potential outcomes.

**Results:**

The qualitative responses were coded into nine leadership behavior themes listed here in order from most to least often mentioned: Kindness, Supportive, Open to Input, Allow Autonomy, Engage with Team, Transparency, Fairness, Professionalism, Hold Accountable. All behavior themes related significantly to trust of the leader, with three themes relating significantly to job satisfaction (Transparency, Fairness, and Professionalism).

**Discussion:**

These results provide a more specific view of leader behavior than does the typical style approach.

## 1 Introduction

Most leadership research and theory focus on characteristics that make successful leaders and/or on effective leadership behavioral styles. In his Annual Review article, Carton (2022) pointed out that our understanding of leader characteristics is more advanced than our understanding of how they lead. He further suggests that the domination of leadership styles, which are conglomerates of empirically related behaviors, has serious weaknesses and should be abandoned. Such a broad-based approach fails to clearly identify specific behaviors that can be followed by leaders as they require a leader to figure out exactly how to enact a particular style. He calls for “a wholesale break from the past” (p. 84) by focusing on more specific leader behaviors that are not aggregated into broad styles. To that end we conducted a qualitative study of leadership in which we invited direct reports of managers to describe their leader’s behavior. Text analysis enabled us to derive a series of themes that reflects more specific behaviors than frequently studied styles (e.g., authentic, ethical, transactional, or transformational leadership). Our study offers a glimpse into how followers view their managers when asked to describe their leadership behaviors.

### 1.1 Focus on leader behavior rather than style

A leadership style is a composite of related behaviors, sometimes dozens, that reflects a specific category of leader behavior such as authentic (Avolio et al., 2004), ethical (Brown et al., 2005), servant (Canavesi and Minelli, 2022), or transformational (Bass, 1999). Most studies ask followers to rate their leaders on items reflecting one or more styles, with those styles being developed largely from theories about what constitutes good leadership. For example, authentic leadership had its origins in transformational leadership (Avolio et al., 2009) and servant leadership was developed conceptually, based largely on a philosophy of how leaders should treat followers (Canavesi and Minelli, 2022).

Carton (2022) noted limitations of this approach, including that not all leaders perform all behaviors in a style, that it limits our understanding of the interplay of the behaviors, and that ratings of different behaviors likely suffer from halo error with followers rating consistently across items regardless of their leader’s actual behavior. There is considerable literature on follower cognitive processes, both explicit beliefs and implicit theories, that affect their perceptions of leadership style (Oc et al., 2023). With these limitations in mind, Carton (2022) called for new leadership research that begins fresh without being anchored in past decades of style research. This would enable us to avoid misspecifications by focusing on important behaviors that might or might not co-occur within individual leaders. Those behaviors could then be linked to important attitudinal and behavioral outcomes in followers.

### 1.2 Effects of leaders on follower experience

Leadership theories generally (Carton, 2022) and specifically in healthcare (Ferreira et al., 2022) suggest that a leader’s behavior and style have an impact on important outcomes, including follower attitudes and feelings about their leader (e.g., Judge et al., 2004; Hoch et al., 2018). Leaders set the tone for a work group and have disproportionate influence over the working environment that affects follower experiences of work. Two potential outcomes of trust in the leader and job satisfaction have been particularly relevant in this domain as they have been prominent in research across many different leadership theories (Banks et al., 2016; Hoch et al., 2018). Furthermore, different leadership models suggest that trust is a proximal reaction to leadership style that can lead to the more distal outcome of job satisfaction.

Trust of the leader is defined as follower expectations about the leader’s intentions concerning them (Dirks and Ferrin, 2002). A trusted leader is seen as someone who is safe to be open with, and who will have one’s best interest at heart. Job satisfaction is a work attitude that is thought to be heavily influenced by leader behavior and style. Meta-analyses of many different styles (e.g., authentic, ethical, servant and transformational) show consistent relationships with trust (Rai and Kim, 2021; Lux et al., 2023) and job satisfaction (Canavesi and Minelli, 2022).

### 1.3 The current study

We conducted a multi-method study of leadership to determine how direct reports’ descriptions of their managers’ behavior would relate to the outcomes of trust and job satisfaction. A qualitative approach was used to determine the sorts of leader behaviors that were most salient to our participants (i.e., direct reports) as they reflected on their manager. Each participant completed a survey that asked them to describe their manager’s leadership behavior and then rate their trust in that manager and their own job satisfaction. A content analysis of the open-ended responses identified behavioral themes, and the themes were connected to the quantitative scores. This enabled us to test if participants who described their manager as exhibiting a particular type of behavior (e.g., supportive) would have higher quantitative scores on trust and job satisfaction than those whose descriptions were the opposite (e.g., unsupportive). The study identified specific behaviors that were most salient to direct reports and organized them into narrow themes. It also indicated the extent to which direct report attitudes were related to their experience of these behaviors from their supervisors.

## 2 Materials and methods

### 2.1 Sample and procedure

Our participants for the study comprised 273 direct reports of 44 managers from an academic hospital in the Southeastern United States. The departments were spread throughout the hospital including clinical and nonclinical units. Senior leadership was open to conducting leadership research and engaged university researchers to collaborate in that effort. All 1,383 direct reports of these managers were invited to complete surveys about their manager and their own perceptions and attitudes. An email list of all direct reports from the 44 managers was provided by the human resources department. The survey was conducted over approximately 2 weeks, with an initial email invitation and two follow-up reminders sent to all direct reports. A total of 317 direct reports completed the surveys (18.5%), but 44 were dropped because they did not provide descriptions of leader behavior. Examples of unusable responses included “fine” “Good,” “Not sure of the leadership style” “ok.” The survey was hosted on Survey Monkey.

### 2.2 Measures

#### 2.2.1 Qualitative measure

A single open-ended question appeared at the beginning of the survey after the informed consent and prior to the quantitative items. It was “*Describe how your direct supervisor functions as a leader. How does he/she act towards you and your coworkers?*” A text box allowed participants to write in a response. Length varied with some writing long explanations and others listing a few keywords. We asked the qualitative question first to minimize potential biases that might occur from answering quantitative items that serve to prime open-ended answers. Both team members who did the coding were Americans with a similar educational background and who have done leadership research. This might have affected their coding and produced results different from individuals from different cultural backgrounds and with different research experiences.

#### 2.2.2 Quantitative measures

We chose well-established scales with established psychometric properties. Trust of the leader was measured with a 6-item scale provided by Podsakoff et al. (1990). They report a coefficient alpha for the scale of 0.90, and predictive validity evidence as significant correlations with direct reports job satisfaction, organizational citizenship behavior and perceptions of transformational leadership. Job Satisfaction was measured with the 3-item subscale from the Michigan Organizational Assessment Questionnaire (Cammann et al., 1979). Bowling and Hammond (2008) conducted a meta-analysis of 80 studies using this scale and reported a mean coefficient alpha of 0.84 and significant correlations with a wide range of variables expected to relate to job satisfaction. All items used the same 7-point agreement scale with choices ranging from *disagree very much* to *agree very much*. High scores represented high levels of each construct.

### 2.3 Coding process

**Figure 1:**
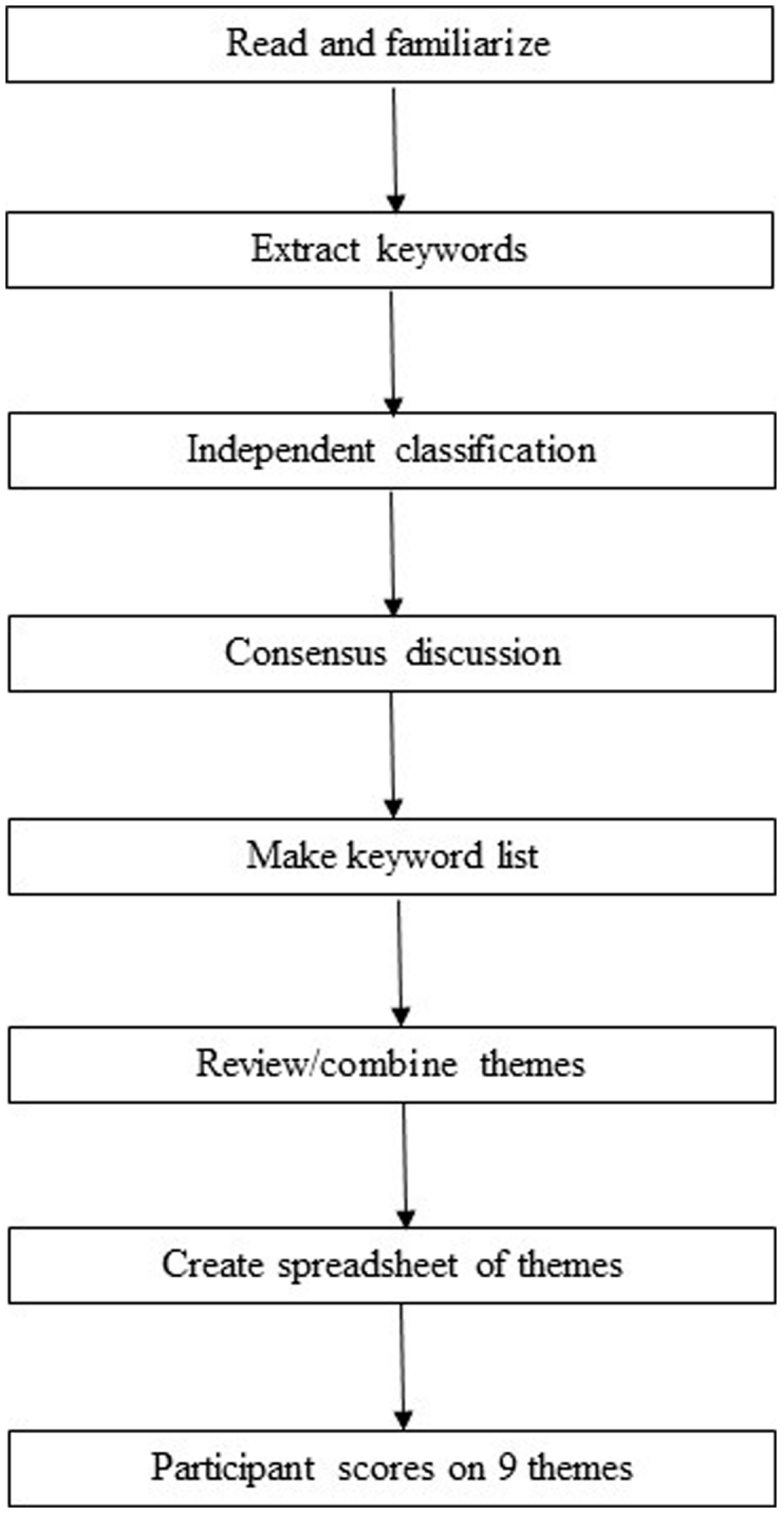
Flow chart of coding procedure.

We began by coding the qualitative results to identify themes, and then for each participant we checked if each of the themes was represented in their response. We chose a manual coding approach because we wanted to identify specific behaviors within each direct report’s response and one person might mention more than one behavior. We were not interested in the overall themes for each response that would be reflected in combinations of behaviors, which could easily be identified with automated coding using software. Such combinations would reflect more of a broad leadership style. Two members of the team served as coders. Both are experienced researchers who both conduct and teach qualitative methods. The coding was done in an iterative process as follows (see Figure 1 for an overview).

- One member of the team read all responses to become familiar with the content. It was apparent that there was a mixture of positive and negative behaviors noted.
- On a second pass, a list of keywords (single words and short phrases) was extracted that reflected manager behavior.
- The two coders independently classified each keyword as a positive, negative, or neutral (or unclear) behavior by the manager. Positive behaviors are those that would be considered signs of good leadership or treatment of others whereas negative behaviors would be signs of poor leadership and bad treatment of others. To establish inter-rater agreement, we compared the classifications and found an initial 80% agreement. A consensus meeting was held, where the two coders discussed each keyword where there was disagreement. After each person explained their judgment, a consensus was attempted about whether the item was positive or negative. If no consensus was reached, or both agreed that the keyword was ambiguous, the classification was considered neutral. The final list consisted of keywords where there was consensus on the classification as positive or negative.
- The two keyword lists (positive and negative) were examined and placed into initial themes, 10 on the positive and 9 on the negative side.
- The two lists were reviewed and refined. It became apparent that there were nine themes reflected in both lists. The positive list reflected a manager who embodied the theme (e.g., being fair) whereas the corresponding theme in the negative list reflected the opposite (e.g., being unfair). The lists were merged so that each theme was represented at the positive end and the negative end. What we report in the tables is the positive end of each theme, with the negative end being the absence of the theme (e.g., engaged vs. not engaged or supportive vs. not supportive) or the opposite (being fair vs. unfair).
- A spreadsheet was created with themes placed in columns, and direct reports represented as rows. The complete responses for each direct report were reviewed a third time to see which of the nine themes were represented. For each direct report’s row, a 2 indicated that one or more keywords reflected being on the positive end on the associated theme (e.g., democratic) and a 1 meant one or more keywords reflected being at the negative or low end of the theme (e.g., autocratic). If the theme was not represented the cell was left blank.

## 3 Results

### 3.1 Emerging themes

Table 1 lists the nine behavior themes (column 1) and indicates a maximum of 5 keywords as examples for each one, representing that the behaviors comprising the theme were at the high end of the continuum (column 2) versus low (or opposite) end (column 3). For example, with Kindness the keyword “*nice*” represents a manager who is high on kindness, whereas the keyword “*demeaning*” indicated a manager who was at the low end. We chose order of prevalence to reflect potential importance, as some were not often mentioned. We limited keywords to 5 per theme to aid in reading/interpretation of the table. The entire list is available from the first author upon request. The table indicates the percentage of the sample that fell in each theme (column 4); for example, over 40% of direct reports mentioned kindness, but only 3.7% mentioned accountability. The last column is the percentage of times that when the theme was indicated it was at the high end (column 5). For example, about a quarter of the responses reflected the theme of supportive, with 86% of those responses indicating their manager was supportive and 14% that they were not, or in terms of frequencies, 74 direct reports mentioned supportive behavior, with 64 of them at the high end. The percentages in column 4 sum to more than 100% because some participants’ responses reflected more than one theme. Specifically, 45% of the sample’s responses reflected one theme, 40% reflected two themes, 12% reflected 3 themes, 3% reflected four themes and 1% reflected five themes. The themes most likely to occur together were Kindness and Openness (25 times), Kindness and Supportive (25 times), Kindness and Fairness (18 times), Allow Autonomy and Openness (17 times), and Openness and Supportive (16 times).

**Table 1:** Themes, example keywords, and frequency of sample who mentioned each one.

Theme	Behavior present	Behavior not present	Percent of hospital sample	Percent of comments that were at the high end
Kindness	Cares, compassionate, considerate, kind, nice	Condescending, critical, degrading, demeaning, hostile	40.7%	89%
Supportive	Advocates, challenges, encourages, helpful, supportive	Does not advocate, does not stand up for, lack of support, no help, no support	27.1%	86%
Open to input	Approachable, hears all sides, inviting, listens, open	Dismiss concerns, does not listen, does not take suggestions	26.7%	90%
Allow autonomy	Collaborative, consultive, democratic, empower, not a micromanager	Authoritarian, autocratic, bureaucratic, micromanage, does not allow autonomy	21.6%	80%
Engage with team	Available, hands on, involved, present	Absent, distant, hard to reach, little presence, unavailable	15.8%	40%
Transparency	Communicative, explains, informative, sets example, transparent	Not transparent, poor communicator	13.9%	76%
Fairness	Fair, inclusive, treats as equals	Biased, does not treat the same, favoritism, preferential treatment, unfair	15.4%	62%
Professionalism	Ethical, honest, integrity, professional, trustworthy	Lies, passive/aggressive, twist the truth, unprofessional	10.2%	89%
Hold accountable	Holds accountable	Conflict avoidant, does not enforce rules, no accountability	3.7%	30%

Percentages sum to more than 100% because some followers indicated more than one theme. Maximum of five example behaviors shown.

Table 2 indicates for each theme the percent of the entire sample that was high (column 2) versus low (column 3). For example, 36% of the entire sample of 273 gave a response that reflected that their manager was kind, whereas 4% indicated their manager was unkind.

**Table 2:** Percentage of sample mentioning each theme as present or absent.

Theme	Present	Absent
Kindness	36	4
Supportive	23	4
Open to input	24	3
Allow autonomy	17	4
Engage with team	6	10
Transparency	11	3
Fairness	10	6
Professionalism	9	1
Hold accountable	1	3

*n* = 273; Present percentages sum to more than 100% because some followers indicated more than one theme.

### 3.2 Description of the themes

#### 3.2.1 Kindness

The most often mentioned theme, indicated by more than 40% of the sample, was Kindness. This reflects the extent to which managers treat employees in a caring and compassionate way, and often the keyword kindness itself was mentioned. The vast majority of participants (89%) who mentioned kindness did so in a positive way. For example, one indicated that their manager was, “*very kind, concerned and friendly*.” A small minority (4% of the entire sample) indicated that their manager was unkind or treated direct reports in a critical and even hostile manner; an example is, “… *never has anything nice to say. Always negative and demeaning*.”

#### 3.2.2 Supportive

More than a quarter of the sample mentioned that their manager was (or was not) supportive, that is, they provided instrumental help in doing the job, and/or emotional support. We distinguished supportive from kindness as it reflected behavior that was helpful but not necessarily in a kind way. Furthermore, a manager might have been described as nice, but passive and not particularly supportive. Out of the 74 participants whose responses fit the supportive theme, only a third also described their manager as kind. A supportive manager was in some cases described as an advocate, “*…fiercely advocates for our unit on all issues that arise.*” Others described their manager as helpful, often including the word ‘supportive’, as in the following example, “…*extremely helpful around the unit and with problem solving. She addresses situations on the unit immediately and is always very supportive of her employees.*” A small minority of participants mentioned a lack of support, for example, “*I feel like I do not have a manager to stand up for me, and really have no resource for support.*”

#### 3.2.3 Open to input

This theme concerned upward communication and the extent to which a manager solicited and was open to input. A positive example is, “*Friendly, approachable, asks if we have any issues to discuss at our monthly meetings*”; a negative example is, “*Standoffish, unapproachable*.”

#### 3.2.4 Allow autonomy

This theme distinguished democratic from autocratic managers. A democratic manager is collaborative and consultive, whereas an autocratic manager is an authoritarian or a micromanager. A democratic manager was described as, “*…has a democratic leadership style. When problems arise, the team is expected to participate in creating and implementing solutions*.” An autocratic manager was described as, “*Authoritarian leadership, the direction comes from above and it is directed for us to follow the direction without input.*”

#### 3.2.5 Engage with the team

Engaged managers are those who are interact with their direct reports and are seen as available and hands on with their unit. An engaged manager talks to direct reports whereas an unengaged manager might stay in their office and avoid interacting. One participant described their manager as, “*informative, approachable if help needed and engaged*.” At the other end, a disengaged manager was described as, “…*distant and unavailable.*”

#### 3.2.6 Transparency

This theme had to do with downward communication and the willingness of a manager to keep direct reports informed. One participant described a transparent manager as, “*…always transparent and explains procedures in detail*.” An opposite type of manager was described as, “*poor communicator with getting information to everyone and not one select group of people.*”

#### 3.2.7 Fairness

This theme involves fairness in how direct reports were treated and whether or not there was favoritism. A fair manager was described as, “*…very fair and impartial with our interactions*.” An unfair manager was characterized as, “*Biased. Unfair. Plays favorites.*”

#### 3.2.8 Professionalism

Ethics, honesty, and integrity comprised this theme. A manager embodying professionalism was described as, “*uses direct communication and is open and honest*.” A manager viewed as unprofessional was described as, “*Very unprofessional…Lied about the shift.*”

#### 3.2.9 Hold accountable

An accountable manager enforces rules and standards appropriately. An participant described an accountable manager as, “*She is compassionate and understanding to me. I feel like she holds me accountable.*” One participant described a manager who struggles with accountability as, “*Does not enforce rules, has actually told me “I cannot hold everyone accountable”…wants to be friends and not a leader.*”

### 3.3 Mixed method analysis

Descriptive statistics (means, standard deviations, and coefficient alphas) were computed for each of the two quantitative measures. Trust had a mean of 33.0, a standard deviation of 9.1, and coefficient alpha of 0.88. Job satisfaction had a mean of 17.7, standard deviation of 4.2, and coefficient alpha of 0.81. Trust and job satisfaction were significantly correlated (*r* = 0.44, *p* < 0.05).

The qualitative and quantitative data on outcomes were linked. We had coded each theme as being negative (1) or positive (2). We computed correlations between this theme “score” and each of the four quantitative outcomes using a value of *p* of 0.05. If a theme was not mentioned by a follower, it was considered as missing data for these analyses. Thus, the sample size for each analysis was the number of participants who used keyword descriptors for that theme. Table 3 contains the results. The second column indicates the sample size (n) for each theme, ranging from 10 to 111. Despite the rather small sample sizes for some of the themes, Trust had a statistically significant correlation with every theme, and these correlations are quite large. These results suggest that those participants who say their manager embodies each of these themes have greater trust as indicated by the quantitative data. Job satisfaction had smaller correlations than trust for all but professionalism and was statistically significant only for the themes of Professionalism, Fairness, and Transparency.

**Table 3:** Correlations among themes and outcomes.

Theme	*N*	Trust of leader	Job satisfaction
Kindness	111	0.70^*^	0.15
Supportive	74	0.68^*^	0.15
Open to input	73	0.72^*^	0.19
Allow autonomy	59	0.49^*^	0.03
Engage with team	43	0.72^*^	0.27
Transparency	38	0.90^*^	0.44^*^
Fairness	42	0.86^*^	0.44^*^
Professionalism	28	0.71^*^	0.84^*^
Hold accountable	10	0.84^*^	0.44
Positivity	273	0.76^*^	0.28^*^

* *p* < 0.05.

We computed a positivity index that combined results across themes as the percentage of themes represented in a response that were positive. Thus, a zero means that all themes represented were negative, whereas 100 means all themes represented were positive. Numbers in between indicate both positive and negative themes were represented, e.g., 50 means an equal number of each. The last row of the table shows statistically significant correlations between the positivity index and the two outcomes with trust having the larger correlation. Thus, participants who viewed their managers in a generally positive way had significantly greater trust in that manager and higher job satisfaction than counterparts who described their managers in negative terms.

## 4 Discussion

We answered Carton’s (2022) call to start fresh in exploring foundational leadership behaviors that were more specific than broad styles. We chose to investigate leader behavior from the perspective of direct reports and how they describe their managers’ behaviors. This taps into those salient acts that are top of mind when followers reflect on their leaders. This approach may say as much about followers as leaders as it provides a snapshot of explicit beliefs and perceptions that are not necessarily important for leaders’ overall effectiveness. Because we were starting from scratch, we chose to use a qualitative approach that provided unconstrained descriptions from followers. Furthermore, we wanted to determine how each of the styles would relate to trust and job satisfaction, which are considered outcome variables in most leadership theories.

We found nine behavior themes that reflected categories that for the most part are narrower than the styles that are prominent in the literature. Some of these themes are reflected in broader established styles, but often several of our themes are found in a single existing style. For example, looking just at the more modern values-based theories, Openness and Transparency are clearly represented in the popular 4-dimension conception of authentic leadership (Walumbwa et al., 2008), Openness, Fairness, Professionalism, and Hold Accountable are represented in Ethical Leadership (Brown et al., 2005), and Support, Autonomy and Hold Accountable are features of Servant Leadership (van Dierendonck and Nuijten, 2011). Openness, Engagement and Professionalism are characteristics of Transformational leadership while Hold Accountable and lack of engagement characterize Transactional leadership (Avolio et al., 1999).

### 4.1 Interpersonal treatment

The two most often mentioned themes in our study were Kindness and Supportive, both of which have to do with the quality of interpersonal treatment received. Although many of our participants described leaders who embodied (or failed to embody) both, the majority did not, providing evidence that people see these as distinct. Some managers might be very caring and nice but provide limited help and support to direct reports. This is important because there is growing awareness that merely being kind does not necessarily provide effective support, which is an important component of leadership (Gray et al., 2022). Kindness can be seen in some of the newer leadership theories that focus on interpersonal treatment of followers (Banks et al., 2021), most notably servant leadership theory (Kuonath et al., 2021), although a close examination of items used to assess these types of leadership do not have items that explicitly reference kindness as described by our participants. Furthermore, an entire area of leadership study has focused on the antithesis of kindness, most notably abusive supervision (Tepper, 2000), and we saw keywords that connote such treatment, such as “*critical*,” “*degrading*,” and “*demeaning*.”

Research and theory about support has perhaps come more from the occupational stress domain than leadership, viewing support as important for coping with difficult situations (Viswesvaran et al., 1999). It is a core component of Leader-Member Exchange (LMX) theory (Scandura and Schriesheim, 1994) that focuses on relationship quality between leaders and followers.

It should be kept in mind that these results come from an American sample, and there are likely cultural differences in how people from other countries view interpersonal treatment. For example, Americans and Chinese can differ in how they handled interpersonal conflicts at work (Liu et al., 2008).

### 4.2 Communication

Whereas Kindness and Support have to do with the tone of communication, Open to Input and Transparency concern content and flow of communication. A leader who is open creates an upward flow of information, seeking opinions from followers. Such a leader is described as approachable and inviting. A transparent leader promotes a downward flow by sharing information and keeping followers informed of what is going on and the reasons for decisions. Leaders who are transparent are described as being good communicators. Although some of our participants described their leaders as having both of these qualities, many mentioned only one of them, suggesting a one-way flow in some cases. Some leaders might make themselves a central hub in a one-way communication flow, either collecting information from followers without sharing, or providing information and instructions while remaining indifferent to follower input. In both cases the use of only a single direction can create communication problems within a work unit by isolating management from direct reports. A flow only downward limits the feedback and information a manager has about what is happening in their unit, which can lead to poor and uninformed decisions. A flow only upward leaves direct reports uncertain about how their manager views their actions. A lack of such feedback can undermine motivation as direct reports are uncertain about expectations and feel isolated from the organization.

Managing two-way communication flow in a work unit can be challenging for managers. Being open to upward communication invites comments on decisions made by the manager, and if not well managed can give direct reports the impression that they get to approve all decisions. Managers need to maintain clear boundaries, making it clear that inviting input does not guarantee that a decision will be dictated by direct reports. It is important that direct reports know that their input is valued, even in cases where the manager does not agree and fails to base decisions on direct report input.

### 4.3 Managing people

The remaining themes reflect different aspects of people management. Allow Autonomy is a concept that can be found throughout the leadership literature as a means of empowering and motivating employees. It is reflected in styles that are characterized as democratic or participative. It is seen at least as far back as the Ohio Leadership Studies as reflected in the Tolerance and Freedom subscale of the Leader Behavior Descriptive Questionnaire XII (Stogdill, 1963), and it can be seen as one of the four major styles in Path-Goal Theory (House, 1996). The idea that employees can be motivated by empowering them and allowing autonomy is supported by self-determination theory’s suggestion that autonomy is one of the three basic human needs (Deci and Ryan, 2009). It should not be surprising that the concept of autonomy has played a prominent role in the broader organizational literature. For example, autonomy is an important element in the stress process, and its lack can be considered a stressor that relates to both physical (Nixon et al., 2011) and psychological (Spector et al., 1988) strains.

Autonomy can be important in the high-stakes environment of healthcare that relies heavily on teamwork. Often individuals have limited personal autonomy over how and when they do specific tasks, for example, during surgery people’s roles and tasks are highly routinized. Even in this setting, however, it is possible for people to be given autonomy within boundaries, and it certainly can be represented by allowing individuals to make suggestions and ask questions. It can also be manifest by allowing individuals latitude in the tasks they take on, even if how those tasks are done is proscribed, and in their scheduling.

An aspect of managing people that has not been prominent is seen in the Engage with Team theme. Our participants talked about the extent to which leaders were available and present, with some talking about a disengaged leader. Such a leader might be what is described by transactional leadership theory as passive (Avolio et al., 1999), but it is possible for a leader to be engaged with a team by interacting on a daily basis yet be indecisive when it comes to taking actions. Alternatively, a disengaged leader might be quite active and have no problem making decisions but does so in isolation and rarely interacts with followers. A disengaged manager leaves a vacuum by providing limited leadership. Thus, direct reports are left to manage themselves, which can produce a breakdown in teamwork since there is no leader to coordinate and direct efforts.

The literature on fairness and justice is immense but goes well beyond treatment by direct leaders. Measures of fairness ask about overall treatment, with interactional justice being perhaps most closely linked to leader behavior. However, items of common scales talk about general treatment from superiors rather than a particular leader like the direct supervisor. Nevertheless, the experience of fairness has been strongly linked to a number of important follower variables, so it is not surprise that it correlated significantly with trust in the leader and job satisfaction (Cohen-Charash and Spector, 2001).

Several of our participants talked about the professionalism or lack thereof of their manager. Most used the keyword ‘professional’ or ‘unprofessional,’ but a few made comments about ethics and integrity. Perhaps this is a particular issue in healthcare where ethics and professionalism are important when it comes to patient care.

The idea that supervisors do or do not hold followers and others accountable for their actions can be seen in several leadership theories, although from different perspectives. From an ethical leadership perspective, the behavior reflects a leader who enforces ethical behavior in followers (Brown et al., 2005). For the transactional leader, accountability is manifest as rewards for engaging in desired versus punishments for engaging in undesired behavior (Avolio et al., 1999).

### 4.4 How themes linked to job satisfaction and trust

The strongest and most consistent connection between our themes and outcomes was for trust of the leader. This is not surprising as trust can be a potential outcome that is proximal due to it being a manifestation of how people feel about their leaders. Dirks and Ferrin (2002) proposed a model of trust that includes several variables that reflect, at least in part, our themes, including transformational leadership, support, justice, and participation. They supported these connections empirically with a meta-analysis of survey studies, but our findings provide support using a different methodology.

The correlations of leadership with job satisfaction were in most cases considerably smaller than with trust, and they were not consistent across themes. The smaller correlations likely indicates that follower job satisfaction is less sensitive to leadership than the literature would suggest, as satisfaction can be affected by a wide range of work factors. Nevertheless, it is possible that some behaviors are more important for job satisfaction than are others, or that the potential impact depends on context. For example, in healthcare, professional issues among clinical care employees are extremely important, so it is perhaps not surprising that professionalism (i.e., ethical behavior and integrity of leaders) was strongly related to job satisfaction. It seems that Fairness, Professionalism, and Transparency are potentially important factors in job satisfaction. Fairness is not surprising, considering the large number of studies on fairness in general being linked to job satisfaction (Cohen-Charash and Spector, 2001). Nor is transparency surprising given meta-analytic evidence linking authentic leadership to both attitudes (Banks et al., 2016).

### 4.5 Limitations and future directions

There are four limitations to this study that should be considered. First, although open-ended responses and ratings are drawing on different kinds of judgments, there is still the possibility for biases that cross methods. For example, it is possible that there is an overall likability bias in that direct reports who personally liked their managers were inclined to say only positive things and give positive ratings about the workplace. This would not necessarily affect the themes they chose to include but would have affected which end of the theme was represented, and perhaps inflated correlations between themes and the quantitative measures. Future research should incorporate other methods to assess leadership that does not rely on direct report judgments. This might be done, for example, by content analyzing messages (verbal and written) leaders provide to direct reports.

Second, although the overall sample size was not small, the numbers endorsing each theme were in all but one case less than 100, and in many cases substantially less. This limited our ability to draw confident conclusions about which themes were most important when it came to our two outcomes as the power to detect significant differences and to achieve precise effect size estimate was insufficient for that purpose. Clearly larger samples would be needed to make comparisons among themes in relationships with our two outcomes as well as others. Alternatively, a quantitative study could be conducted in which direct reports are asked to rate their managers on scales representing our nine themes, as well as trust, job satisfaction, and other potential outcomes.

Third, data were collected in a single organization representing a single industry. It is conceivable that direct reports in other hospitals or industries would describe different behaviors. Although most of our themes would seem to reflect universal behaviors that would be relevant across industries, professionalism might be more salient in healthcare than other industries.

Fourth, it is possible that there were biases in the coding of the open-ended responses. Both coders were experienced leadership researchers, and that might have influenced how they viewed the descriptions of behavior. Bias was mitigated to some extent by having initial coding done independently. A second mitigating factor was that the consensus discussion had no mandate to come to an agreement, and in cases where the coders did not see keywords in the same way, they were relegated to the neutral/ambiguous category.

This study provides only a brief snapshot of leader behavior from the perspective of followers. As with all survey studies relying on employee reports, it is not clear the extent to which reports of leader behavior are accurate, and if interventions that manipulate leadership will have expected effects on direct reports. In other words, will results from survey studies on direct reports generalize when we conduct experimental studies involving interventions. Moving forward we need to incorporate other perspectives and other methods. For example, leaders themselves could be asked to provide self-descriptions of their behavior, either via open-ended questionnaires or interviews. Observers could shadow a sample of leaders for some period of time, making note of leader behaviors and the apparent impact on followers. Finally, our nine themes could be operationalized as rating scales that could be used for self-report by leaders, or other-reports by followers. A 360-degree approach could be applied by combining ratings from direct reports, peers, and the target leader’s supervisor. A quantitative approach would be useful in determining co-variation among behaviors and whether distinct profiles of behaviors exist, as well as relative strength of relationships with non-leadership variables at the employee and organizational level. This could answer questions such as the optimal leader behavior profile that drives employee engagement or organizational results.

### 4.6 Concluding thoughts

We answered Carton’s (2022) call to revisit the behaviors that followers observe in their leaders. We identified nine behavior themes that were apparent when direct reports were asked to describe their manager’s leadership behavior. These themes are more specific than the broad styles found in the literature that can cut across a wide variety of behaviors. Our themes can be found sprinkled throughout the leadership literature, embedded in broader styles that define various leadership types. What is unclear is how the themes might co-exist within leaders and the relative importance of these themes for follower outcomes. The nine themes identified here might represent a first step in redefining leadership behavior types. Rather than describing leaders in broad styles as authentic or transformational, we might describe them in terms of specific behavior patterns, such as accountable leaders, engaged leaders, kind leaders, or transparent leaders. This is more in line with Carton’s (2022) argument to focus more on specific behaviors and less on broad styles. Research moving forward might take a quantitative approach in assessing these nine themes in the behavior of leaders and seeing how they relate to important outcomes. It might also identify clusters of behaviors that tend to co-exist that could provide a taxonomy of leaders based on their behavior combinations.

From a practical perspective, our results suggest targets for leadership training that focuses on specific behaviors rather than general styles. Rather than asking leaders to be more authentic or more transformational, our results suggest more specific advice such as ask for direct report opinions, encourage, hold accountable, and listen to all sides. Trainers can explore how to do each of these, offering advice and opportunities to practice.

Our qualitative results have provided a glimpse into how followers view leader behavior, but more importantly have raised new questions about the best dimensions along which to study leadership, and how best to describe the complex set of behaviors that constitute leadership.

## Data availability statement

The raw data supporting the conclusions of this article will be made available by the authors, without undue reservation.

## Ethics statement

The studies involving humans were approved by University of South Florida, Institutional Review Board. The studies were conducted in accordance with the local legislation and institutional requirements. The ethics committee/institutional review board waived the requirement of written informed consent for participation from the participants or the participants’ legal guardians/next of kin because project was approved as exempt.

## Author contributions

PS: Conceptualization, Formal analysis, Methodology, Writing – original draft. DH: Conceptualization, Methodology, Writing – review & editing. EE: Conceptualization, Methodology, Writing – review & editing. JC: Conceptualization, Methodology, Writing – review & editing. JQ: Conceptualization, Formal analysis, Methodology, Writing – review & editing.
